# An iteration normalization and test method for differential expression analysis of RNA-seq data

**DOI:** 10.1186/1756-0381-7-15

**Published:** 2014-08-13

**Authors:** Yan Zhou, Nan Lin, Baoxue Zhang

**Affiliations:** 1 Institute for Genomic Biology, University of Illinois at Urbana-Champaign, Urbana, IL 61801, USA; 2 Key Laboratory for Applied Statistics of MOE and School of Mathematics and Statistics, Northeast Normal University, Changchun, 130024 Jilin Province, P. R. China; 3 Department of Mathematics, Washington University in Saint Louis, 63130 Saint Louis, Missouri, USA

**Keywords:** RNA-seq, Normalize, Expression level, TMM, IMM

## Abstract

**Background:**

Next generation sequencing technologies are powerful new tools for investigating a wide range of biological and medical questions. Statistical and computational methods are key to analyzing massive and complex sequencing data. In order to derive gene expression measures and compare these measures across samples or libraries, we first need to normalize read counts to adjust for varying sample sequencing depths and other potentially technical effects.

**Results:**

In this paper, we develop a normalization method based on iterating median of M-values (IMM) for detecting the differentially expressed (DE) genes. Compared to a previous approach TMM, the IMM method improves the accuracy of DE detection. Simulation studies show that the IMM method outperforms other methods for the sample normalization. We also look into the real data and find that the genes detected by IMM but not by TMM are much more accurate than the genes detected by TMM but not by IMM. What’s more, we discovered that gene UNC5C is highly associated with kidney cancer and so on.

## Introduction

Deep DNA sequencing methods (ChIP-seq and RNA-seq) offer distinct advantages in increased specificity, sensitivity and genome-wide comprehensiveness that are leading to their wider use. It has been showed that splicing variants [1,2] and single nucleotide polymorphisms [3] can be detected through sequencing the transcriptome, opening up the opportunity to interrogate allele-specific expression and RNA editing.

The reads produced by RNA-Seq are first mapped to the reference genome using computer programs. Then, the output of RNA-Seq can be summarized by a sequence of ‘counts’. That is, for each gene, it gives a count standing for the number of reads whose mapping starts at that gene. As different libraries have different total read counts, i.e., sequencing depths. In order to compare the genes expression and detect distinction between libraries, we should normalize the libraries. The aim of normalization is to remove systematic technical effects that occur in the data, and ensure that technical bias has minimal impact on the results. Experience with microarray data showed that normalization is a critical component of the processing pipeline, allowing accurate estimate and detection of DE genes [4]. However, the procedure for generating RNA-seq data is fundamentally different from that for microarray data, the normalization methods used in microarray data are therefore not directly applicable in RNA-seq data.

Current RNA-seq analysis methods typically standardize data between samples by scaling the number of reads in a given library to a common value across all sequenced libraries in the experiment. Several researchers have modeled the observed counts for a gene with a mean that includes a factor for the total number of reads [5-7]. Similarly, for LONGSAGE- seq data, t Hoen et al. [8] used the square root of scaled counts, Vencio et al. [9] proposed a beta-binomial model to normalization. Mortazavi et al. [10] adjusted their counts to reads per kilobase per million mapped (RPKM). Cloonan et al. [11] log-transformed the gene length-normalized count data and applied standard microarray analysis techniques (quantile normalization and moderated t-statistics).

Here, we search a better normalization procedure which focus on two main questions: (1) Does the normalization improve DE detection (sensitivity) in reducing the false discover rate. (2) Does the normalization result in low technical variability across replicates (specificity)? The standard procedure is to compute the proportion of each gene’s reads relative to the total number of reads, and compare that across all libraries, either by transforming the original data or by introducing a constant into a statistical model. Robinson et al. [12] proposed a scale normalization (TMM) method which is two-side symmetry trimmed log-fold-changes. Compared to the previous normalization, the method shows improved results for inferring differential expression in simulated and real data. But the TMM method can not normalize the data reasonable when the data are asymmetric, especially, when the proportion of DE genes is large (Additional file 1: Figure S1). Exclusion of most of genes may lead to lost of too much information and the TMM normalization scale is estimated by a symmetry trimmed will bring biased results when the data are asymmetric. We develop a new method with an iteration median of M-values (IMM) to normalize the samples of different sequence depths. The IMM method normalizes the libraries without a symmetry trimmed. The aim of iteration process of IMM method is to look for an invariant set of non-DE genes and use the invariant set to normalize the samples.

The rest of this paper is organized as follows. In Section ‘Results and discussion’, we introduce the TMM normalization method and propose an iteration normalization method (IMM) for detecting DE genes. We carry out extensive simulation studies in Section ‘Simulation studies’. In Section ‘Application to real dataset’, we illustrate our method by analyzing a liver and kidney dataset. Finally, some conclusions are drawn.

## Results and discussion

### Sampling framework

The following framework is a formal explanation for the requirement of normalization. Let *Y*_*gk*_ and *μ*_*gk*_ be the observed count summarized from the raw reads and true mean expression level (number of transcripts) for gene *g* in library *k*, respectively. *L*_*g*_ as the length of gene *g* and *N*_*k*_ as the total number of reads for library *k*. We can model the expected value of *Y*_*gk*_ as:

$$E\left[Y_{\text{gk}}\right]=\frac{\mu_{\text{gk}}L_{g}}{S_{k}}N_{k},\;\;\;\text{where}\;S_{k}=\overset{G}{\underset{g=1}{\sum }}\mu_{\text{gk}}L_{g},$$

*S*_*k*_ represents the total RNA output of the *k*th sample. The underlying problem for the analysis of RNA-seq data is that while *N*_*k*_ is known, *S*_*k*_ is unknown and may vary among different samples, and depend on the RNA composition.

### The trimmed mean of M-values normalization method

The total RNA production, *S*_*k*_, cannot be estimated directly, since the expression levels and true lengths of every gene is unknown. However, the relative RNA production of two samples, $f_{\text{kr}}=\frac{S_{k}}{S_{r}}$, essentially a global fold change, can be easily determined. Define the gene log-fold-changes for sample *k* relative to sample *r* for gene *g*;

$$M_{\text{gk}}^{r}=\log_{2}\frac{Y_{\text{gk}}N_{r}}{Y_{\text{gr}}N_{k}},$$

and absolute expression levels;

$$A_{\text{gk}}^{r}=\frac{1}{2}\log_{2}\left(\frac{Y_{\text{gk}}}{N_{k}}\times \frac{Y_{\text{gr}}}{N_{r}}\right)\;\;\text{for}\;Y_{\text{g}\cdot }\neq 0.$$

A trimmed mean is the average value after removing the upper and lower *x%* of the data. The TMM procedure is doubly trimmed, by log-fold-changes $M_{\text{gk}}^{r}$ and by the absolute expression level $A_{\text{gk}}^{r}$. The suggested trimming proportion in Robinson et al. [12] is $M_{\text{gk}}^{r}$ values trimmed by 30*%* and the $A_{\text{gk}}^{r}$ values by 5*%* is a robust TMM factor. After trimming, the TMM method takes a weighted mean of $M_{\text{gk}}^{r}$, with weights as the inverse of the approximate asymptotic variances (calculated using the delta method [13]). Specifically, the normalization scaling factor ${\text{TMM}}_{k}^{\left(r\right)}$ for sample *k* using reference sample *r* is calculated as:

$${\text{log}}_{2}({\text{TMM}}_{k}^{\left(r\right)})=\frac{\underset{g\in G*}{\sum }\frac{M_{\text{gk}}^{r}}{w_{\text{gk}}^{r}}}{\underset{g\in G*}{\sum }\frac{1}{w_{\text{gk}}^{r}}},$$

$$\text{where}\;\;w_{\text{gk}}^{r}=\frac{N_{k}-Y_{\text{gk}}}{N_{k}Y_{\text{gk}}}+\frac{N_{r}-Y_{\text{gr}}}{N_{r}Y_{\text{gr}}},\;\;Y_{\text{gk}},\;Y_{\text{gr}}>0.$$

The cases where *Y*_*gk*_ = 0 *orY*_*gr*_=0 are trimmed in advance of this calculation since log-fold-changes cannot be calculated; G* represents the set of genes with valid $M_{\text{gk}}^{r}$ and $A_{\text{gk}}^{r}$ values after trimmed with the above percentages. Then Robinson et al. [12] apply the TMM normalization factor ${\text{TMM}}_{k}^{\left(r\right)}$ to detect DE genes.The TMM normalization can normalize the samples well when the log-fold-changes are symmetry (Additional file 1: Figure S1). However, when the log-fold-changes are asymmetric, the two side symmetry trimmed may be unreasonable (Additional file 1: Figure S2).

### The iterated median of M-values normalization method

We propose a robust normalization procedure that reduces the bias of estimation without introducing additional noises. We propose computing the log-fold-changes by excluding the DE genes. In this paper, we complete the normalization with hypothesis test, and search a normalization factor by iterating, then use the normalization factor to detect the DE genes.

Under two conditions, if DE genes do not exist, the expression level of gene will be equal between libraries (*μ*_*g*1_ = *μ*_*g*2_) across all genes and the log-fold-changes ($M_{\text{gk}}^{r}$) are concentrated around zero. Hence we should use all genes to calculate the normalization factor. However, when DE genes exist, it is unreasonable if we still use all genes to normalize the libraries. The DE genes confuse the log-fold-changes of the non-DE genes when the count of DE genes are used in the total number of reads. Ideally, we should use only non-DE genes for normalization. The IMM normalization factor is the median of fold changes of remaining genes. 

(1) Step one 

a. Define *Y*_*gk*_ as the observed count for gene *g* in library *k*. In this step, all genes are used for normalization. We use the median of log-fold-changes ($M_{\text{gk}}^{r}$) as the normalization scaling factor of sample *k* using reference sample *r*, which is calculated as

$${\text{log}}_{2}({\text{IMM}}_{0k}^{r})={\text{median}}_{g\in G}M_{\text{gk}}^{r},$$

where ${\text{IMM}}_{0k}^{r}$ is the initial normalization scaling factor. *G* represents the set of genes excluding these with *Y*_*gk*_ = 0 or *Y*_*gr*_ = 0. It should be clear that ${\text{IMM}}_{0r}^{r}=1$. For a two-library comparison, the scaling factor is a one-dimensional scale. But for technical replicates, normalization factors across several libraries can be calculated by selecting one sample as a reference and calculating the IMM factor for each non-reference library, and then obtain a scale vector ${\text{IMM}}_{0}=({\text{IMM}}_{01}^{r},{\text{IMM}}_{02}^{r},\ldots ,{\text{IMM}}_{0k}^{r})$.

b. We use the normalization scaling factor *IMM*_0_ to calculate *P*-*values* of all genes. For two libraries, we use an amended sage.test function from the CRAN statmod package [14] to compute a Fisher exact *P*-*value* for each gene. We replace the original total number of reads for library with the ‘effective’ total number of reads. The effective total number of reads for library is calculated by multiplying/dividing the square root of the estimated normalization factor with the sum count of remain genes of library. For technical replicates, we follow the analysis procedure used in the Marioni et al. study [5]. We use following two methods to calculate the *P*-*values* of genes. The first method is an exact Poisson statistic. Assume that the counts mapping to a gene are Poisson-distributed. That is,

$$Y_{\text{gk}}∼\text{Pois}(\lambda_{{\text{gz}}_{k}}{\text{IMM}}_{0k}^{r}),$$

where *z*_*k*_ is the experimental condition of library *k* and $\lambda_{{\text{gz}}_{k}}$ represents the fraction of total reads for gene *g* in experimental condition *z*_*k*_. The total and group total counts are all Poisson distributed. Then the two-sided *P*-*value* is the sum of all the probabilities that are less than or equal to the observed probability. The second method is LR testing [5]. We fitted the Poisson GLM model first, computing the maximum likelihood estimates under both the null and alternative hypothesis. The standard likelihood ratio statistic, was computed, and *P*-*values* were obtained using the fact that, under the null hypothesis, this statistic has a *χ*^2^ distribution with 1 degree of freedom.

c. Following Benjamini and Hochberg [15], we adjust *P*-*values* to correct for multiple testing. All the genes that are tested for significance are ranked by their *P*-*values*. Then for each gene, the *Q*-*value* is given by

$$Q-\text{value}=P-\text{value}\times \frac{\text{count}}{\text{rank}}$$

where *count* is the total number of genes tested and *rank* is the rank of the *P*-*values*. If *Q*-*value* is less than 0.005, note this is only a threshold and it does not represent the FDR for overall procedure, we call the genes difference. We determine genes $g_{01},g_{02},\ldots ,g_{0i_{0}}$ as DE.

(2) Step two

We exclude the genes $g_{01},g_{02},\ldots ,g_{0i_{0}}$ which are determined DE in Step one, normalize the samples with the remaining genes, and obtain the normalizing scaling factor *IMM*_1_. Repeat b and c in Step one with the remaining genes and the scaling factor *IMM*_1_, and determine DE genes $g_{11},g_{12},\ldots ,g_{1i_{1}}$.

(3) Step three

Repeat Step two until ${\text{IMM}}_{(j+1)k}^{r}={\text{IMM}}_{\text{jk}}^{r}=\text{IMM}$. Then $g_{(j+1)1},g_{(j+1)2},\ldots ,g_{(j+1)i_{\left(j+1\right)}}$ are treated as the final set of DE genes, and *IMM* is our estimated scaling factor.

(4) Step four

We apply the final scaling factor *IMM* to the samples and use the same test method in 1.b to calculate *p*-*values* of all genes. Then same as in 1.c, we use the BH procedure to claim DE genes at a given FDR level.

The IMM use the median fold-changes as the normalization scaling factor which is more robust than the weighted mean of log-fold-change. What’s more, the iterated excluding the DE genes may be more reasonable than the two side symmetry trimmed the log-fold-changes when the log-fold-changes are biased and the rate of DE genes is large.

## Simulation studies

To investigate the performance of the IMM normalization method, we run simulations to study the effects of RNA composition on DE analysis of RNA-seq data and compare with the TMM method. We include parameters for the number of genes expressed uniquely to each sample, and parameters for the proportion, magnitude and direction of differentially expressed genes between samples. The simulation is set up to sample a dataset from a given empirical distribution of read counts (that is, from a distribution of observed *Y*_*g*_). The mean is calculated from the sampled read counts divided by the sum *S*_*k*_ and multiplied by a specified library size *N*_*k*_ (according to the model). The simulated data are then randomly sampled from a Poisson distribution with a given mean. Since we have inserted known differentially expressed genes, we can rank genes according to various statistics and plot the number of false discoveries as a function of the ranking.

To start, we simulate from just two libraries. We introduce two libraries data with 10*%* unique-to-group expression for the first condition, 5*%* or 50*%* DE at a 4-fold level, 90*%* of which is higher under the first condition. (Additional file 1: Figure S1) and (Additional file 1: Figure S2) show M versus A plots for a typical simulation including unique genes and DE genes and indicate the normalization effects of the IMM normalization and the TMM normalization. We consider different rates of DE genes, and compare the two normalization scales. As we can see that, TMM and IMM perform similarly when the proportion of DE genes is about 10*%* (Additional file 1: Figure S1). However, when the proportion increases to 50*%*, the IMM normalization is obviously closer to the center of non-DE genes than the TMM normalization (Additional file 1: Figure S2).

Next, we compare the normalization and test methods by the false discovery rate (FDR) curve of different numbers of selected genes. Additional file 1: Figure S3 ∼ Additional file 1: Figure S8 show false discovery plots amongst the genes that are common to both conditions, where we have introduced 10*%* unique-to-group expression for the first condition, and 5*%*, 10*%*, 20*%*, 30*%*, 40*%* and 50*%* of DE genes at a 4-fold level respectively, 90*%* of which is higher in the first condition. We observe from Additional file 1: Figure S3 ∼ Additional file 1: Figure S8 that the FDR of IMM normalization method is lower than that of the TMM normalization method as the rate and the bias of DE genes increase. Obviously, the IMM normalization is more robust than TMM method.

To further compare the performance of the IMM normalization with the TMM method and previously used methods in the context of the DE analysis of RNA-seq data, we extend the above simulation to include replicate sequencing runs. Specifically, we compare seven published methods: length-normalized count data that have been log transformed and quantile normalized, as implemented by Cloonan et al. [11]; a Poisson regression [5] with library size; a Poisson regression with TMM normalization [12]; a Poisson regression with IMM normalization; a Poisson exact test [7] with library size; a Poisson exact test with TMM normalization and a Poisson exact test with IMM normalization. We do not directly compare the normalization to virtual length [1] or RPKM [10] normalization. In this paper, the virtual length of genes is generally absorbed into the expression level parameter and does not get used in the inference procedure. However, Sultan [1] used the virtual length of gene to calculate the *q*-*value* of each gene. The formal of RPKM [10] is

$$\text{RPKM}=\frac{Y_{\text{gk}}}{L_{\text{gk}}N_{k}}\times 10^{9},$$

where the define of *Y*_*gk*_,*L*_*gk*_and*N*_*k*_ are same as above. If the virtual length of gene *L*_*gk*_ is absorbed into RPKM, the normalization is the same as the total library size normalization. The simulation condition is the same as the above simulation just with two replicates. We made the simulation data Poisson-distributed to mimic technical replicates. Additional file 1: Figure S9 and Additional file 1: Figure S10 show false discovery plots amongst the genes with different rates of DE genes. Among the methods (Poisson likelihood ratio statistic, Poisson exact statistic), the same normalization method performance is very similar. It can be seen that the IMM normalization method has much lower false discovery rate than other methods as the rate of DE genes increases.

In additional simulation studies, we fixed two of the three parameters and see the curve of FDR versus to the rest parameter. The three parameters are the proportion, magnitude (fold) and direction (offset) of differentially expressed genes between samples, respectively. It is shown that if the DE genes are symmetry, the FDR of three normalization methods are little different (Additional file 1: Figure S13). However, when there are obviously offset, the FDR of the IMM method is lower than the other methods (Additional file 1: Figure S13). The Additional file 1: Figure S14 give a result that the IMM method is better than the other methods with the proportion of differentially expressed genes increasing. Additional file 1: Figure S15 ∼ Additional file 1: Figure S18 show that the TMM and IMM methods are both much better than the library size normalization with the fold of differentially expressed genes between samples increasing. As the proportion increase, the IMM method preform better than the TMM method (Additional file 1: Figure S18).

## Application to real dataset

### A liver versus kidney data set

In this section, we apply our method to a publicly available transcriptional profiling data set comparing several technical replicates of a liver and kidney RNA source [5]. Human housekeeping genes, as described in [16], were downloaded from [17] and matched to the Ensembl gene identifiers using the Bioconductor [18] biomaRt package [19]. The real data has been analyzed by Robinson et al. [12]. The distribution of M values (liver to kidney) is skewed in the negative direction, therefore the library size normalization is not fit to the real data. Since there obvious exist bias, the TMM normalization trim data symmetry and remove most of genes including 421 of 538 housekeeping genes (Additional file 1: Figure S11), it therefore may be unreasonable. On the contrary, the IMM normalization may be more accurate which only removes 246 of 538 housekeeping genes. After the IMM trimmed, the log-fold-changes of the remain genes is concentrated around zero, which are calculated by the counts of genes divided by the total counts of the remain genes (Additional file 1: Figure S11).

The application of IMM normalization to this pair of samples results in a normalization factor of 0.989 (-0.016 on *log*_2_ scale; shown by the red line in Additional file 1: Figure S12) after excluding some genes. The IMM normalization is a robust method from the simulation studies and the factor is robust for bias data where more DE genes on one hand may be expected. When the false discovery rate (*q*-*value*) is no more than 0.0001, we call gene differentially expressed between liver and kidney. We use the exact Poisson test to detect the DE genes with differential normalization method and obtain the number of called DE genes in Table 1. We compare the IMM method with the TMM normalization and the library size normalization. Using IMM normalization in a statistical test for DE, the ratio of genes significantly higher in liver (or kidney) is similar to that using the TMM normalization. The number of housekeeping genes called DE (329) with IMM normalization is similar to that of TMM normalization (330). However, the number of total genes called DE (8083) using the IMM method is more than that of the TMM method (8069) (Table 1).

**Table 1:** Number of genes called differentially expressed between liver and kidney at a false discovery rate <0.0001 using different normalization methods

	**Library size**	**TMM**	**IMM**	**Overlap**
	**normalization**	**normalization**	**normalization**	
Higher in liver	2082	3759	3746	2082
Higher in kidney	7496	4310	4337	4292
Total	9578	8069	8083	6374
House keeping				
genes (538)				
Higher in liver	40	121	118	40
Higher in kidney	357	209	211	207
Total	397	330	329	247

A thorough comparative evaluation of identified differentially expressed gene list is challenging due to the difficulty of defining a gold standard. However, public RNA-seq data set generated in the same tissues in other studies would provide some insights into the performance of our method. Therefore, we downloaded lung and kidney RNA-seq data form bodymap project [20]. DE genes (only protein coding genes were considered) detected by IMM were intersected with DE genes detected by TMM and DE genes only detected by one method were retrieved. Then, we compared the gene expression of these genes in liver and kidney based on bodymap data. Of the 4 liver protein coding DE genes identified only by IMM, 1 has higher expression level in liver than in kidney. Of the 42 kidney protein coding DE genes identified only by IMM, all 42 genes have higher or same expression level in kidney than in liver.On the contrary, only 4 out of 26 TMM-specific liver DE genes have higher expression in liver than in kidney and 16 out of 26 have higher expression level in kidney. Of the 4 kidney DE identified only by TMM, 2 have higher expression in kidney than in liver. Therefore, DE genes detected by IMM are more consistent with gene expression level reported by bodymap data than DE genes detected by IMM.

In addition, we examine the DE genes detected by IMM but not by TMM and their associated diseases. The disease hypoproteinemiais is associated with gene B2M, which is one of the 18 liver genes detect by IMM. A mutation in this gene has been shown to result in hypercatabolic hypoproteinemia (provided by RefSeq, Sep 2009). We also investigate the 45 kidney genes with higher expression and find that gene UNC5C is highly related with kidney cancer ([21-23]). UNC5C has a direct association with kidney cancer. Therefore, IMM detects some important genes which not detected by TMM.

### Other datasets

We also analysis other datasets with the different normalization methods. Here, we first download the dataset [11] which is comparing mouse embryoid bodies versus embryonic stem cells, sequenced on the SOLiD system. The number of genes is 19005, and approximately 500 “housekeeping” genes (using summaries from [24]) are used in the example. There are only two samples which without technical replicates, hence we use the amended sage.test function to calculate *P*-*value* for each gene. The estimated TMM scaling factor is 1.04 and the IMM scaling factor is 1.02. The TMM normalization trim 365 of 495 housekeeping genes, but the IMM normalization only trim 178 of 495 housekeeping genes. (Additional file 1: Table S1) and (Additional file 1: Table S2) take the false discovery rate (*q*-*value*) is no more than 0.0001 and 0.000001, respectively. In Additional file 1: Table S1, both TMM and IMM methods better than Library size normalization method with *q*-*value* no more than 0.0001. When the FDR threshold reduced to 0.000001, the number of genes called DE using the IMM method is same as that of the TMM method in housekeeping genes (Additional file 1: Table S2). However, the IMM method discovers 8347 DE genes of all genes, which is more than 8243 of the TMM method (Additional file 1: Table S2).

Another example concerns human embryonic kidney (HEK) and Ramos B cells RNA source [2]. These samples are also without technical replicates. The total number of DE and housekeeping genes are in Additional file 1: Table S3, with q-value no more than 0.0001. Additional file 1: Table S3 shows that the IMM method is better than the TMM method, but worse than the library size normalization method.

## Conclusions

Normalization will be crucial in many other applications of high throughput sequencing where the DNA or RNA populations being compared differ in their composition. Similar to previous high throughput technologies such as microarrays, normalization is an essential step for inferring true differences in expression between samples. The number of reads for a gene is dependent not only on the gene’s expression level and length, but also on the population of RNA from which it originates. We present a straightforward and effective empirical method for normalization of RNA-seq data.

The IMM normalization is an effective and robust method for estimating relative RNA production levels from RNA-seq data. The IMM method estimates scale factors between samples that can be incorporated into currently used statistical test methods for DE analysis. In our experience, the iterate will converge in no more than five steps. In essence, both microarray and TMM normalization assume that the majority of genes, common to both samples, are not differentially expressed. Our simulation studies indicate that the IMM method is robust under the assumption that the rate of DE genes is no more than 0.5.

The IMM use the median fold-changes as the normalization scaling factor is a robust method. As the TMM normalization method, the IMM also trim some genes as DE genes. But the IMM method is not just simply symmetry trimmed up and down side log-fold-changes. The iterated excluding the DE genes may be more reasonable than the TMM method when the log-fold-changes are biased and the rate of DE genes is large.

From the simulation results, IMM normalization is more robust than TMM. In order to investigate IMM normalization in real data, we use different normalization methods while the same test method to detect DE genes. And we find that DE genes detected by IMM are more consistent with gene expression level reported by bodymap data than DE genes detected by IMM. What’s more, we find DE genes identified only by IMM are more likely related to liver or kidney tissue than DE genes detected by TMM. Therefore, IMM normalization method is a useful method in RNA-seq data analysis for biologists.

## Code

Scripts for our analysis have been wrote in R, which should install the edgeR package [25] in version 2.5 of Bioconductor [18] before running code.

## Competing interests

The authors declare that they have no competing interests.

## Authors’ contributions

YZ, NL and BZ built the model, done the simulations and drafted the manuscript. YZ look into real data and do some biology analysis. All authors read and approved the final manuscript.

## Supplementary Material

Additional file 1 Supplementary tables and figures Click here for file
