# Erratum: An iteration normalization and test method for differential expression analysis of RNA-seq data

**DOI:** 10.1186/s13040-014-0030-4

**Published:** 2014-12-12

**Authors:** Yan Zhou, Nan Lin, Baoxue Zhang

**Affiliations:** Institute for Genomic Biology, University of Illinois at Urbana-Champaign, Champaign, USA; Department of Mathematics, Washington University in Saint Louis, St Louis, USA; Key Laboratory for Applied Statistics of MOE and School of Mathematics and Statistics, Northeast Normal University, Changchun, P. R. China

## Erratum

Following publication of our paper in *BioData Mining* [1], it came to light that numerous sections of text were similar or identical to a previous publication by Robinson and Oshlack [2]. In extending the method proposed by Robinson and Oshlack, we inadvertently copied text from their article [2]. We acknowledge that these areas of text should have been clearly indicated as quotations or excluded entirely. We sincerely apologize for this oversight.
