# Supplementary material for: An iteration normalization and test method for differential expression analysis of RNA-seq data
Source: BioData Min. 2014 Aug 13;7:15. doi: 10.1186/1756-0381-7-15 (PMC4181730; doi:10.1186/1756-0381-7-15)
Supplement: Additional file 1 — Supplementary tables and figures [file 1756-0381-7-15-S1.pdf]

An iteration normalization and test method for differential expression analysis of RNA-seq data

Additional file

Additional file 1: Table S1-S3
Additional file 1: Figure S1-S18.

Table S1: Number of genes called differentially expressed between embryoid bodies (EB) and embryonic stem cells (ES), at a false discovery rate < 0.0001 using different normalization methods.

	Library size normalization	TMM normalization	IMM normalization	Overlap
Higher in EB	4441	4156	3978	3895
Higher in ES	4854	5172	5428	4854
Total	9295	9328	9406	8749
House keeping genes (495)				
Higher in EB	279	258	243	232
Higher in ES	132	140	161	132
Total	411	398	404	364

Table S2: Number of genes called differentially expressed between embryoid bodies (EB) and embryonic stem cells (ES), at a false discovery rate < 0.000001 using different normalization methods.

	Library size normalization	TMM normalization	IMM normalization	Overlap
Higher in EB	4028	3700	3552	3435
Higher in ES	4228	4543	4795	4228
Total	8256	8243	8347	7663
House keeping genes (495)				
Higher in EB	264	240	218	200
Higher in ES	126	134	157	126
Total	390	374	375	326

Table S3: Number of genes called differentially expressed between human embryonic kidney (HEK) and Ramos B cells, at a false discovery rate < 0.0001 using different normalization methods.

	Library size normalization	TMM normalization	IMM normalization	Overlap
Higher in HEK	4070	3538	3859	3538
Higher in B cells	2561	2975	2728	2561
Total	6631	6513	6587	6099
House keeping genes (525)				
Higher in HEK	154	123	143	123
Higher in B cells	172	212	186	172
Total	326	335	329	295

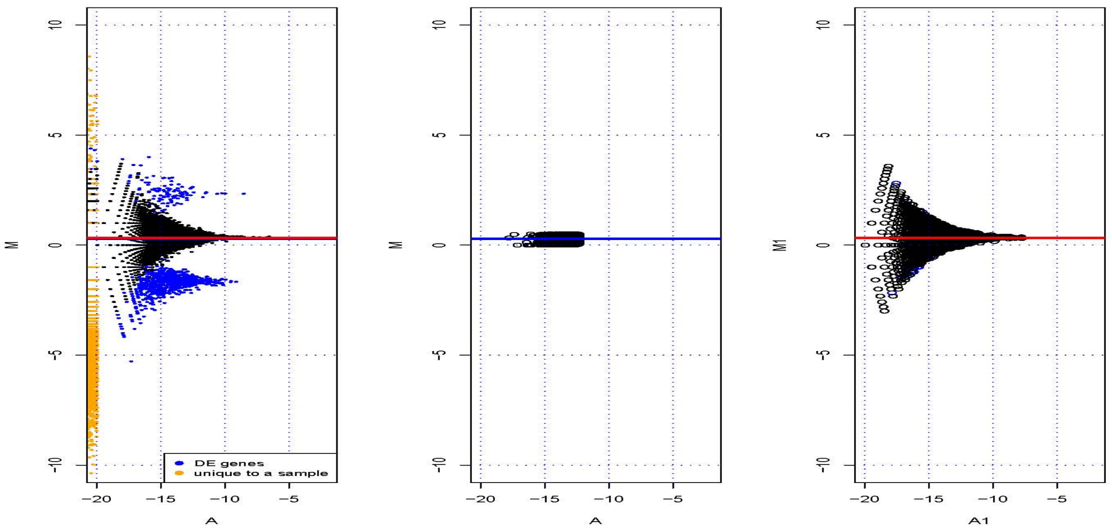

Figure S1: We compare the normalization scales between TMM and IMM method when the rate of DE genes is 10%. The left is M versus A plot of all genes including DE genes and unique genes; The middle is the rest genes and scale (blue line) of TMM normalization; The right is the rest genes and scale (red line) of IMM normalization.

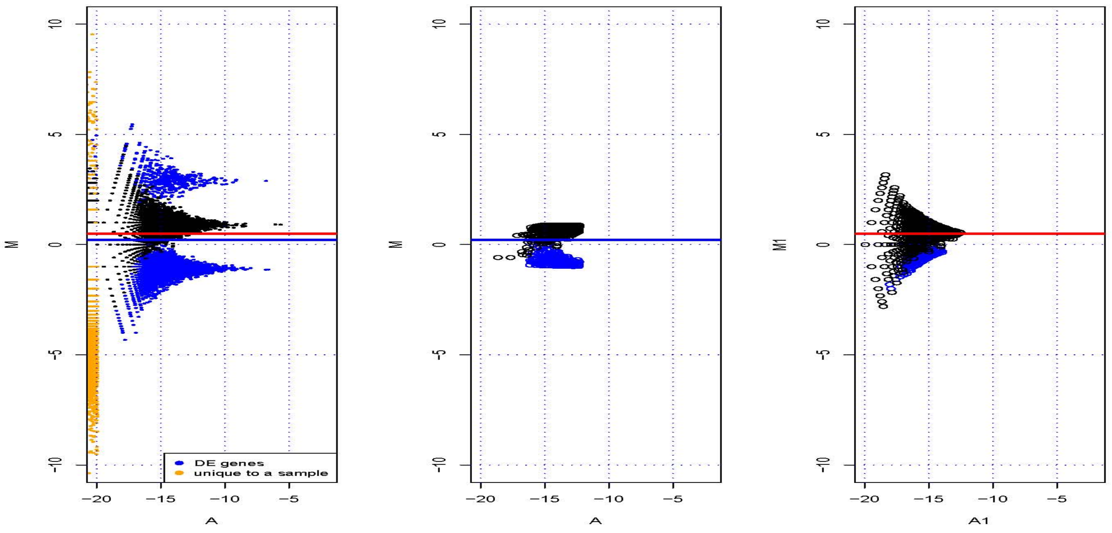

Figure S2: We compare the normalization scales between TMM and IMM method when the rate of DE genes is 50%. The left is M versus A plot of all genes including DE genes and unique genes; The middle is the rest genes and scale (blue line) of TMM normalization; The right is the rest genes and scale (red line) of IMM normalization.

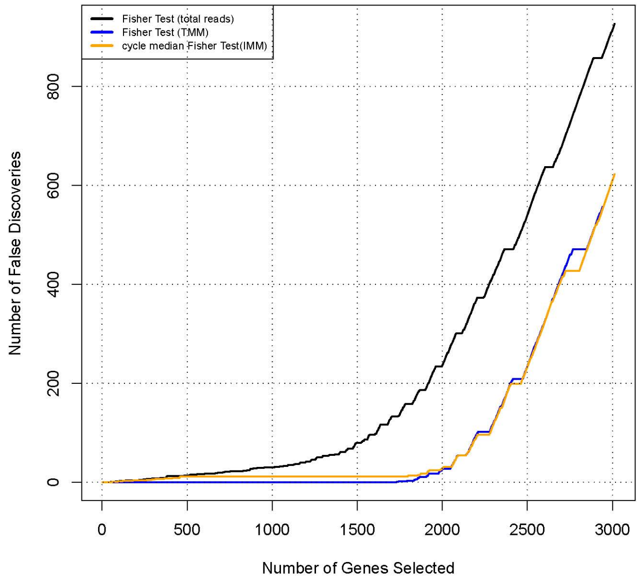

Figure S3: Simulations show IMM normalization is robust and outperforms library size normalization. The plots are the false discovery number of test at the rate 5% DE genes respectively. The black curve is the false discovery number of standard normalization; The blue curve is the false discovery number of TMM normalization; The orange curve is the false discovery number of IMM normalization.

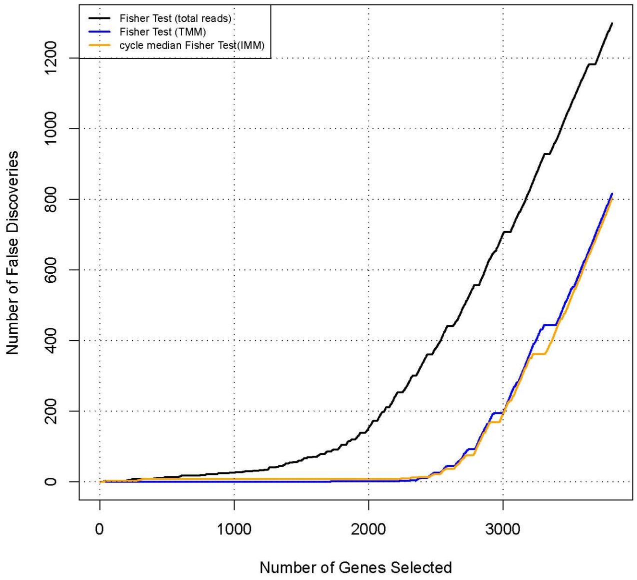

Figure S4: Simulations show IMM normalization is robust and outperforms library size normalization. The plots are the false discovery number of test at the rate 10% DE genes respectively. The black curve is the false discovery number of standard normalization; The blue curve is the false discovery number of TMM normalization; The orange curve is the false discovery number of IMM normalization.

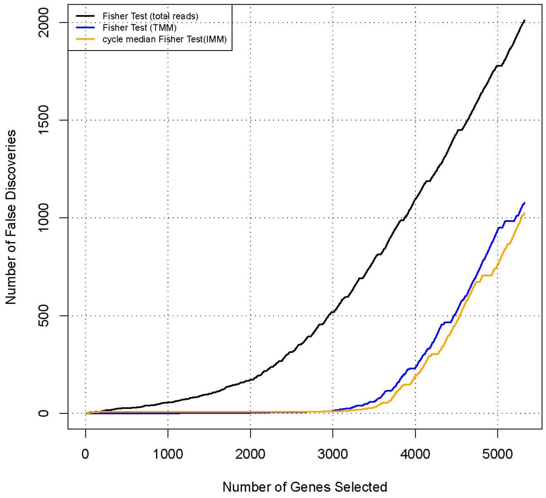

Figure S5: Simulations show IMM normalization is robust and outperforms library size normalization. The plots are the false discovery number of test at the rate 20% DE genes respectively. The black curve is the false discovery number of standard normalization; The blue curve is the false discovery number of TMM normalization; The orange curve is the false discovery number of IMM normalization.

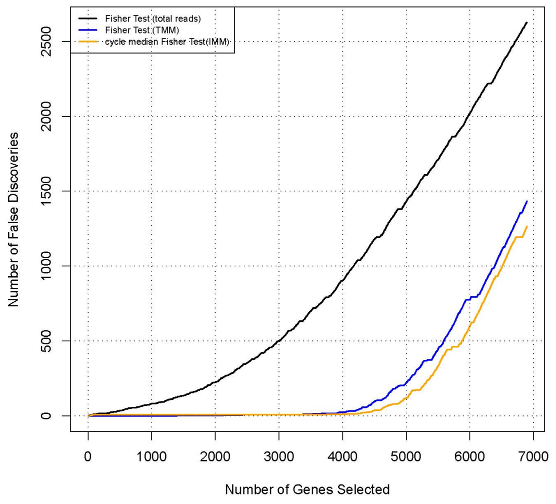

Figure S6: Simulations show IMM normalization is robust and outperforms library size normalization. The plots are the false discovery number of test at the rate 30% DE genes respectively. The black curve is the false discovery number of standard normalization; The blue curve is the false discovery number of TMM normalization; The orange curve is the false discovery number of IMM normalization.

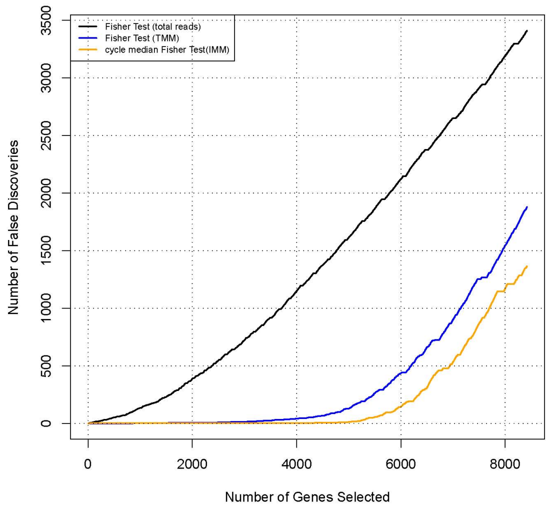

Figure S7: Simulations show IMM normalization is robust and outperforms library size normalization. The plots are the false discovery number of test at the rate 40% DE genes respectively. The black curve is the false discovery number of standard normalization; The blue curve is the false discovery number of TMM normalization; The orange curve is the false discovery number of IMM normalization.

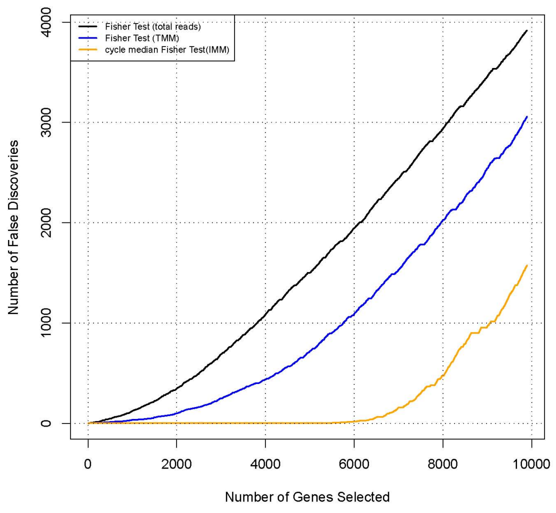

Figure S8: Simulations show IMM normalization is robust and outperforms library size normalization. The plots are the false discovery number of test at the rate 50% DE genes respectively. The black curve is the false discovery number of standard normalization; The blue curve is the false discovery number of TMM normalization; The orange curve is the false discovery number of IMM normalization.

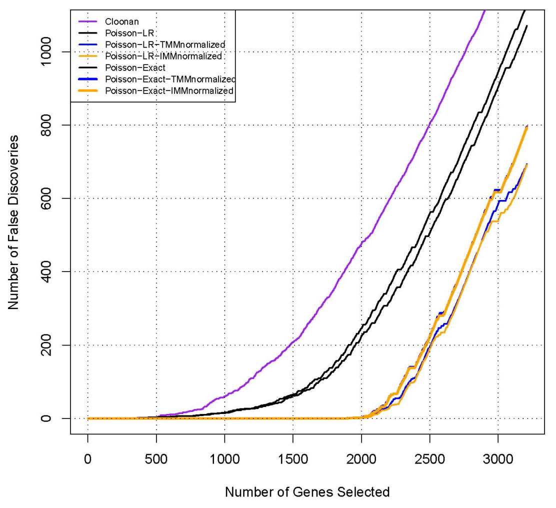

Figure S9: False discovery plots comparing several published methods. The purple line depicts the length-normalized moderated t-statistic analysis. The black lines show the library size normalized; The blue and orange lines show the TMM and IMM normalized analysis, respectively. Figure S9 is the false discovery plots with 10% DE genes.

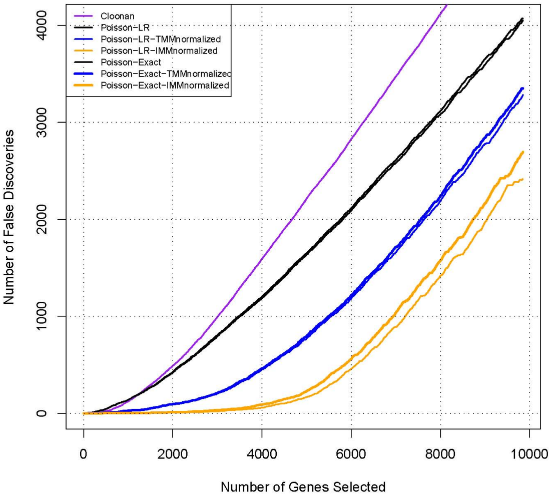

Figure S10: False discovery plots comparing several published methods. The purple line depicts the length-normalized moderated t-statistic analysis. The black lines show the library size normalized; The blue and orange lines show the TMM and IMM normalized analysis, respectively. Figure S10 is the false discovery plots with 50% DE genes.

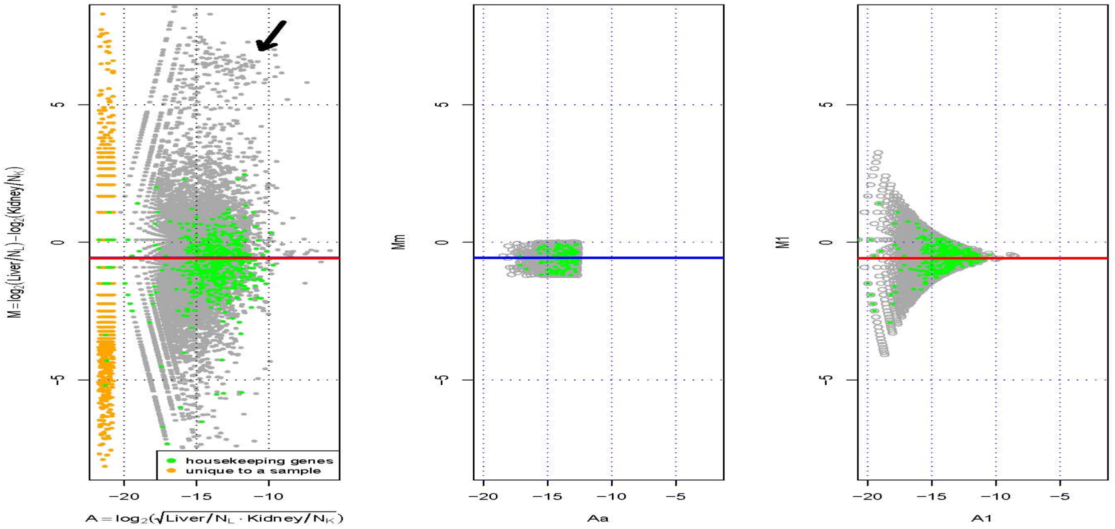

Figure S11: We compare the two normalization between TMM and IMM method. The left is M versus A plot of all genes including DE genes and unique genes; The middle is the rest genes of TMM normalization method; The right is the rest genes of IMM normalization method. The green points are housekeeping genes.

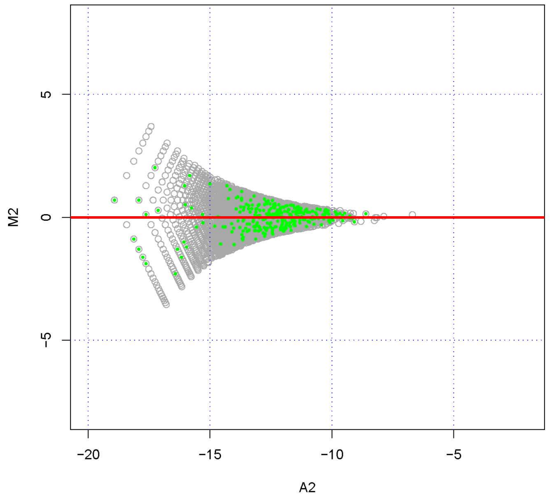

Figure S12: The M versus A and the scale (red line) of rest genes with IMM normalization. Obviously, the M is concentrated around zero and the IMM normalization scale is approximate to zero.

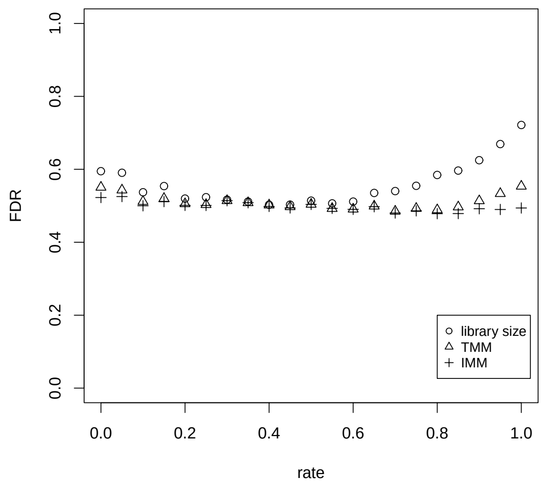

Figure S13: The curve of FDR versus direction of differentially expressed genes, fixed two parameters for the proportion (0.4) and magnitude (4 fold).

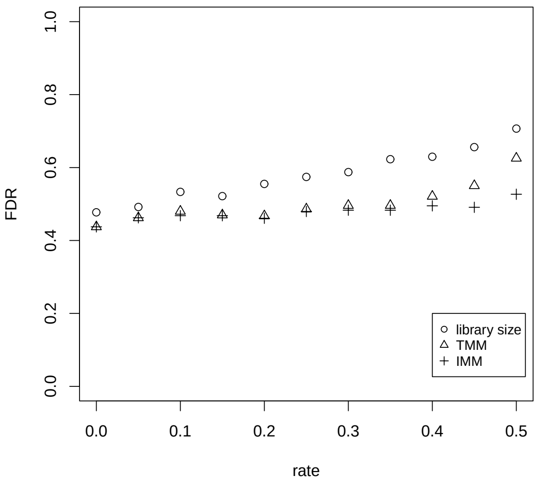

Figure S14: The curve of FDR versus proportion of differentially expressed genes, fixed two parameters for the direction (0.9) and magnitude (4 fold).

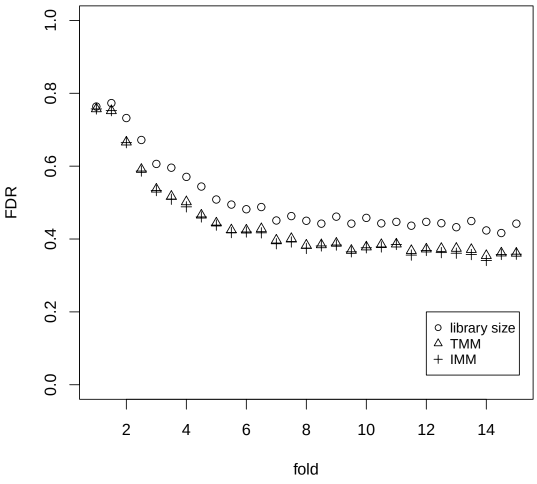

Figure S15: The curves of FDR versus magnitude (fold) of differentially expressed genes, fixed two parameters for the proportion and direction (0.9). The proportion parameter is 0.2

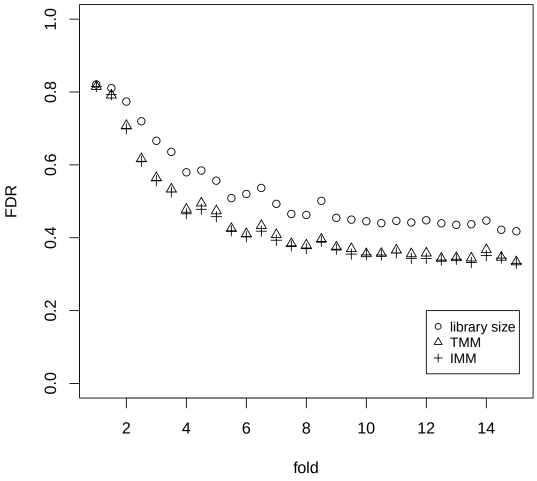

Figure S16: The curves of FDR versus magnitude (fold) of differentially expressed genes, fixed two parameters for the proportion and direction (0.9). The proportion parameter is 0.3

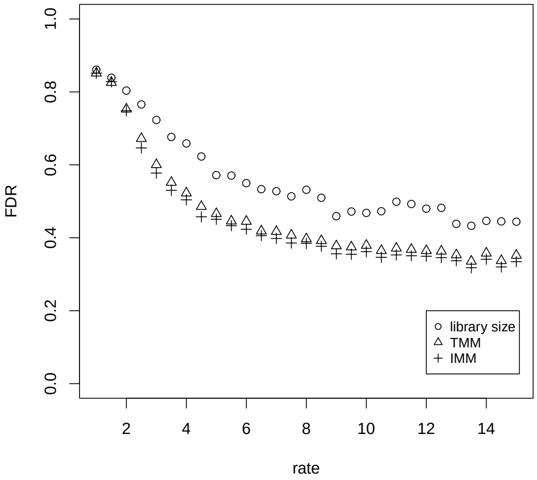

Figure S17: The curves of FDR versus magnitude (fold) of differentially expressed genes, fixed two parameters for the proportion and direction (0.9). The proportion parameter is 0.4

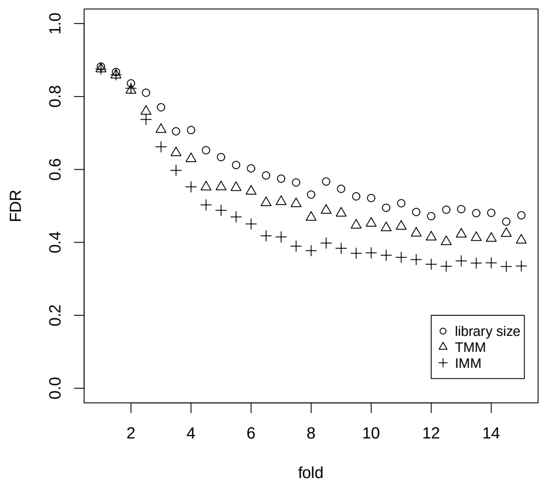

Figure S18: The curves of FDR versus magnitude (fold) of differentially expressed genes, fixed two parameters for the proportion and direction (0.9). The proportion parameter is 0.5.
